# Markers of Immune Cell Exhaustion as Predictor of Survival in Surgically-Treated Early-Stage NSCLC

**DOI:** 10.3389/fimmu.2022.858212

**Published:** 2022-06-27

**Authors:** Laura Sellmer, Julia Kovács, Julia Walter, Jörg Kumbrink, Jens Neumann, Diego Kauffmann-Guerrero, Rosemarie Kiefl, Christian Schneider, Andreas Jung, Jürgen Behr, Amanda Tufman

**Affiliations:** ^1^ Department of Medicine V, Member of the German Center for Lung Research, University Hospital, Ludwig Maximilians University (LMU) Munich, Munich, Germany; ^2^ Department of Thoracic Surgery, Thoracic Oncology Centre Munich, University Hospital, Ludwig Maximilians University (LMU) Munich, Munich, Germany; ^3^ Institute of Pathology, Medical Faculty, Ludwig Maximilians University (LMU) Munich, Munich, Germany; ^4^ German Cancer Consortium (DKTK), German Cancer Research Centre (DKFZ), Heidelberg, Germany

**Keywords:** HAVCR2, immune transcriptomics, NSCLC, TIM-3, immune cell exhaustion

## Abstract

**Background:**

Tumor tissue as well as regional lymph nodes are removed during curative surgery for early-stage non-small cell lung cancer (NSCLC). These tissues provide a unique snapshot of the immune cell composition at the time of surgery. We investigated the immune landscape in matched tumor tissue, tumor bearing (tb) and non-tumor bearing (ntb) N1 as well as N2 lymph nodes (LNs) in patients with NSCLC and its relation to survival.

**Methods:**

Internal hospital databases were screened for surgically treated NSCLC patients for whom tumor tissue, tbLNs as well as N1 and N2 ntbLNs were available. Clinical as well as demographic data were extracted from hospital records. Expression profiling of 770 immune-related genes was performed using the PanCancer IO 360 panel by NanoString Technologies.

**Results:**

We identified 190 surgically treated patients of whom 16 fulfilled inclusion criteria and had sufficient archived tissue. The Tumor Immune Dysfunction and Exclusion (TIDE) score in N1 tumor-free lymph nodes was associated with OS. TIM-3 expression was inversely correlated with TIDE scores in affected LNs, N1 and N2 ntbLNs. Levels of CD8 expression were significantly higher in TIDE High compared to TIDE Low patients. TIM-3 and PD-L1 were selected for the final model for OS in multivariate regression in more than one tissue.

**Conclusion:**

Levels of immune cell exhaustion markers may indicate a dysfunctional immune status and are associated with survival after curative surgery in NSCLC.

## Introduction

Lung cancer is the leading cause of cancer related deaths worldwide (1). Non-small cell lung cancer (NSCLC) is the most common type of lung cancer, making up approximately 85% of cases. In recent years, NSCLC treatment evolved drastically with the introduction of novel radiation techniques, targeted therapies and immunotherapies. However, anatomic resection in early-stage NSCLC remains the preferred curative treatment. While most patients with very early disease are cured by surgical resection, patients with regional lymph node involvement are at higher risk of local or systemic recurrence (2).

Many factors have been shown to impact postoperative survival in NSCLC, such as tumor stage, (neo)adjuvant treatment, extent of surgical resection, perioperative management, postoperative complications or body mass index (3–6). In addition, the importance of the antitumoral immune response has long been recognized in this setting. Recently, the field of immunoncology has gained traction, leading to the introduction of immunotherapies in many entities of cancer such as NSCLC, melanoma, glioblastoma, prostate cancer and others. The introduction of checkpoint inhibitors in NSCLC has led to pronounced improvements in tolerability, progression-free survival and overall survival. The response to these and other immunotherapies (including T-cell therapy, antibody-based therapies or vaccine-based approaches) relies on the immune system’s ability to identify foreign antigens and launch a targeted response. This defense is largely mediated by different T-cell subsets, such as cytotoxic CD8+ T-cells (7, 8).

Activation and invasion of tumor-infiltrating lymphocytes (TiLs) has been shown to be a major determinant of response to checkpoint inhibitor treatment and disease-free as well as overall survival (9). However, if activated T-cells fail to entirely eliminate the tumor, they eventually enter a state of exhaustion. In this state, a persistent exposure to antigen will lead to a dysfunctional state in which cytotoxic T-cells can no longer effectively perform effector functions. T-cell exhaustion is common in cancer as well as some other entities, and leads to disease progression despite competent immune function.

Various scoring metrics to quantify the extent of immune dysfunction in tumors have been developed in order to predict survival or response to checkpoint inhibitor treatment. Differences in their predictive value and included genes reflect the diversity of activation/exhaustion pathways as well as tissue-specific processes. Recently, algorithms such as the Tumor Immune Dysfunction and Exclusion (TIDE) were developed that can be applied to a variety of tissues (10). While it has become evident the extent of tumor immune evasion and dysfunction is crucial for predicting survival, it is as of yet unclear whether this also extends to the state of the immune system in tumor bearing and tumor-free lymph nodes.

Various drugs to prevent or revert T-cell exhaustion are currently under development, for example in the phase III COSTAR trial (using Cobolimab, a TIM3 inhibitor) in advanced NSCLC (ClinicalTrials.gov Identifier: NCT04655976) or in the phase III RELATIVITY-047 trial (using Relatlimab, a LAG3 inhibitor) in metastatic melanoma (11).

In this study we examined differences in the immune landscape in matched tumor tissue, affected and unaffected lymph nodes in patients with NSCLC with a focus on markers of immune cell exhaustion and their relation to patient survival.

## Methods

### Patient Cohort and Tissue Collection

Patients were identified retrospectively from hospital records. We screened records for the following inclusion criteria: histologically-confirmed diagnosis of NSCLC (adenocarcinoma, squamous cell carcinoma or large cell carcinoma), anatomical resection with curative intent, availability of FFPE-embedded tissue material and at least three years of follow-up. We also documented gender, age at diagnosis, survival, and tumor (stage and histology) and treatment details (type of surgery and (neo)adjuvant therapy). All patients were treated for NSCLC at the LMU Klinikum in Munich, Germany between 1999 and 2019.

We obtained approval from the institutional ethics board (reference number: 12-16) and obtained informed consent from all participants. This study was conducted in accordance with the Declaration of Helsinki.

### Tissues and Transcriptomics of Immune-Related Genes

We obtained FFPE-embedded tissues of primary tumor, tumor-bearing lymph nodes (tbLN), and N1 and N2 non-tumor-bearing lymph nodes (ntbLNs) from pathology archives. FFPE blocks containing tumor (primary tumor and lymph nodes, respectively) were identified from pathology reports and reviewed by a senior pathologist based on hematoxylin and eosin stainings. Total RNA was extracted using RNEasy FFPE kits (Qiagen, Hilden, Germany) according to manufacturer’s instructions.

We performed gene expression analysis of 770 genes using the nCounter PanCancer IO 360 panel run on a NanoString FLEX platform (including Prep Station and Digital Analyzer) (NanoString Technologies, Seattle, USA). This panel is specifically designed to target genes involved in tumor microenvironment and immune evasion. The NanoString nCounter platform conducts gene expression analysis without amplification steps, therefore being well suited for analysis of potentially degraded RNA extracted from FFPE tissues. Analysis of results including normalization was performed using NanoString’s proprietary nCounter Analysis software.

Immune cell composition in all samples based on mRNA expression data was estimated using CIBERSORTx analysis in Relative mode with LM22 signature matrix (https://cibersortx.stanford.edu/) (12). This algorithm determines the abundances of 22 immune cell populations based on 547 markers.

### TIDE Score

The Tumor Immune Dysfunction and Exclusion (TIDE) score algorithm was originally developed to assess the immune status in tumor tissues to identify patients who may benefit from immunotherapy. We applied it to immune transcriptomics data from tumor, tbLNs, N1 ntbLNs and N2 ntLNs in order to determine whether the state of the immune system could predict postoperative survival in early-stage NSCLC even in the absence of immunotherapy. TIDE scores were calculated using a web-based tool with NSCLC as cancer type and no previous immunotherapy (http://tide.dfci.harvard.edu/). Negative TIDE score values represent the presence of immune evasion markers, whereas positive TIDE scores represent a lack of immune evasion. We categorized patients into TIDE High for patients with a positive TIDE score and TIDE Low for patients with a negative score. Since this analysis included different tissues from the same patient, we investigated whether TIDE scores were consistently positive or negative across tissues.

### Immune Cell Composition and Exhaustion Markers

In addition to the TIDE score, which incorporates several markers independent of cell of origin, we also investigated cell compositions in our four tissues *via* CIBERSORTx. We compared immune cell composition between patients with TIDE High and TIDE Low patients.

Since a large part of the immunosuppressive environment is mediated through exhaustion of immune cells, we investigated levels of immune exhaustion markers and their association with PFS and OS. In order to obtain the most promising candidates we compiled a list of markers of immune cell exhaustion based on a literature search, investigating TIM-3, CD244, CTLA4, PD-1, PD-L1, BTLA, LAG3, EOMES, TIGIT and FOXP3.

### Statistical Analysis

We reported numerical variables as mean with standard deviation and categorical variables as absolute and relative frequencies. We used univariate Cox regression to model the association of TIDE score and OS and PFS. Additionally, we compared OS and PFS between patients with High and Low TIDE score using Kaplan-Meier curves with p-values from log-rank tests. Immune cell composition in TIDE High and TIDE Low patients was non-normally distributed and therefore compared using Mann-Whitney U tests. To identify immune exhaustion markers associated with OS and PFS we used multivariate Cox regression models with forward selection with cut-off values of 0.1 and 0.2 for inclusion and exclusion, respectively. The following variables were used in the forward regression models: Expression levels of TIM-3, CD244, CTLA4, PD-1, PD-L1, BTLA, LAG3, EOMES, TIGIT, FOXP3, age at diagnosis, sex, stage at diagnosis, presence of chronic lung disease and type of (neo)adjuvant therapy. Results are reported as Hazard Ratios (HR).

We compared differences in the 22 cell populations obtained from CIBERSORTx between tumor, affected lymph nodes, N1 and N2 ntbLNs using within-subject ANOVAs.

Correlation between TIDE scores in the different tissues and immune cell exhaustion markers were analyzed using Pearson’s correlation coefficient. Correlation coefficients were classified as moderate for an *r* between 0.5 and 0.7 and strong for an *r* above 0.7.

A threshold of α ≤ 0.05 for significance was applied for all analyses. All analyses were performed in SPSS version 26.

## Results

### Patient Demographics

756 patients with a lung cancer diagnosis who underwent a surgical procedure between 1999 and 2019 were identified in internal hospital databases. Of these, 190 fulfilled all inclusion criteria (histologically-confirmed diagnosis of NSCLC (adenocarcinoma, squamous cell carcinoma or large cell carcinoma), anatomical resection with curative intent and availability of FFPE-embedded tissue material. Most patients who did not meet inclusion criteria were excluded because they did not undergo anatomical resection with curative intent (such as mediastinoscopy or metastasectomy). Of the remaining 190 patients, sufficient tissue for tumor, tbLNs as well as ntb N1 and N2 LNs was available for 16 patients. The vast majority of the 190 patients were excluded because they did not have any tumor-bearing lymph nodes.

The average age at diagnosis was 65.8 ± 9.4 years and 6 (37.5%) of the patients were male. Median follow-up time was 48.5 ± 38.8 months. Clinical characteristics for all 16 patients are displayed in **Table 1**.

**Table 1:** Overview of clinical features of the 16 patients included in this study.

Patient number	Sex	Age at diagnosis [in years]	Smoking status	Pack years	Stage	(Neo)adjuvant therapy	Other chronic lung disease	Histology	PFS [in months]	OS [in months]
1	Male	68	Former	Unknown	IIIB	RCT	No	Adeno	155	156
2	Female	77	Former	Unknown	IIB	None	No	Adeno	38	45
3	Male	81	Former	Unknown	IIIA	RT	No	Adeno	39	100
4	Male	56	Former	Unknown	IIB	CT	No	SCC	55	98
5	Male	79	Active	Unknown	IIB	CT	No	Adeno	38	50
6	Female	58	Former	30	IIIA	CT	Yes	Adeno	59	73
7	Female	66	Active	50	IIB	None	No	Adeno	41	50
8	Female	61	Former	Unknown	IIIB	RCT	Yes	Adeno	4	7
9	Female	69	Former	40	IIB	None	Yes	SCC	27	102
10	Male	56	Active	100	IIIA	RCT	No	SCC	20	47
11	Female	65	Former	80	IIB	None	Yes	Adeno	9	30
12	Female	50	Active	35	IIIA	None	No	Adeno	24	50
13	Male	68	Former	30	IIB	CT	Yes	Adeno	6	19
14	Female	65	Former	50	IIB	RCT	No	SCC	7	21
15	Female	78	Former	Unknown	IIIA	CT	No	Adeno	17	38
16	Female	56	Never	0	IIIA	CT	No	Adeno	0	40

CT, Chemotherapy; OS, Overall survival; PFS, Progression-free survival; RCT, Radiochemotherapy; SCC, Squamous cell carcinoma.

### TIDE Scores in Hilar Lymph Nodes Are Associated With Survival

Interestingly, while tumor TIDE scores were not significantly associated with OS, the TIDE scores measured in N1 ntbLNs were associated with OS (p=0.03, HR=0.59). Additionally, there was a trend towards significance in N2 ntbLNs (p=0.08, HR=0.57).

**Figure 1:**
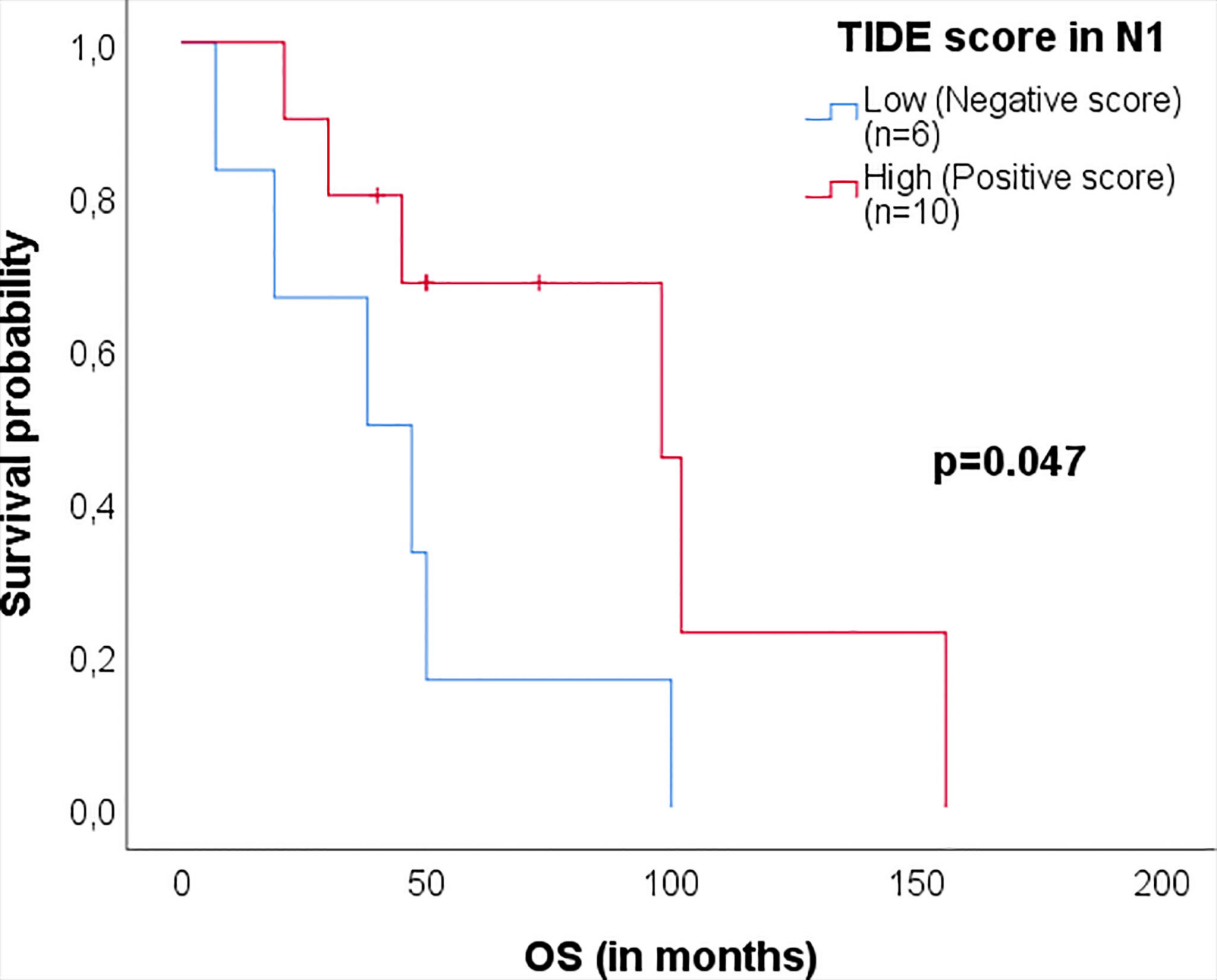
Kaplan-Meier survival plot of patients with TIDE High/Low for OS in N1 ntbLNs. TIDE Low tissues showed negative TIDE values, indicating the presence of immune evasion markers. TIDE High tissues showed positive TIDE values, indicating a lack of immune evasion. P-values were obtained from log-rank tests.

No TIDE score in any single tissue was associated with PFS. All results are displayed in **Table 2** and a Kaplan-Meier survival curve for OS (p=0.05) of TIDE high/low in N1ntbLNs is shown in **Figure 1**.

**Table 2:** Results of univariate Cox regression of the TIDE score in tumor, affected LNs, N1 ntbLNs and N2 ntbLNs.

Tissue	Type of survival	coef	SE	HR	p-value
Tumor	OS	0.31	0.32	1.36	0.33
Affected LNs		-0.24	0.18	0.79	0.20
ntb N1 LN		-0.53	0.24	0.59	0.026
ntb N2 LN		-0.56	0.32	0.57	0.081
Tumor	PFS	0.32	0.28	1.38	0.25
Affected LNs		-0.34	0.2	0.72	0.096
ntb N1 LN		-0.42	0.24	0.66	0.076
ntb N2 LN		-0.52	0.31	0.59	0.093

HR, Hazard Ratio; LN, Lymph node; ntb, non-tumor bearing; OS, Overall survival; PFS, Progression-free survival; SE, Standard error; TIDE, Tumor Immune Evasion and Dysfunction.

Of the 16 patients in this study, only four patients (25%) showed consistently positive or negative scores across all four tissues. Five patients (31%) showed discrepancies in one tissue and the remaining seven (44%) patients showed two positive and two negative scores.

### CD8 Cells Differ Between Patients With Positive and Negative TIDE Scores

When comparing immune cell compositions of all four tissues between patients regardless of TIDE score, we found that M1 macrophages (p=0.02) and neutrophils (p=0.03) had a significantly higher relative abundance in tumor compared to all lymph nodes. In addition, naïve B-cells (p=0.04) and memory B-cells (p=0.004) showed relative lower abundance in tumor tissue compared to lymph nodes. Relative abundances of all cell types are shown in **Supplementary Table 2**.

**Figure 2:**
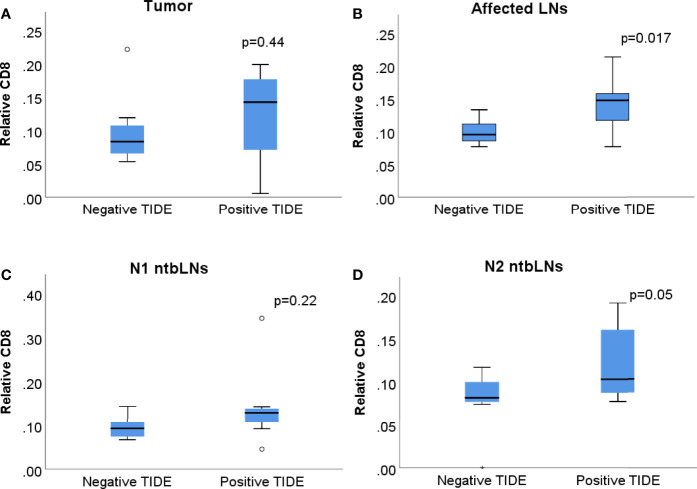
Boxplots of relative abundance of CD8+ cells in patients with negative and positive TIDE scores in **(A)** tumor tissue, **(B)** tbLNs, **(C)** N1 ntbLNs and **(D)** N2 ntbLNs. Boxes show interquartile range, whiskers show lowest and highest quartile.

We then compared patients with positive to patients with negative TIDE scores. CD8 cells were the only cell type with different abundances in patients with positive TIDE scores compared to patients with negative TIDE scores in more than one tissue, with a significantly higher level of CD8 cells in TIDE High patients (**Figure 2**).

### TIM-3 Is Correlated With TIDE Scores and Is Associated With Survival

Since a large part of the immunosuppressive environment is mediated through exhaustion of immune cells, we investigated levels of exhaustion markers and their association with TIDE scores, PFS and OS.

**Figure 3:**
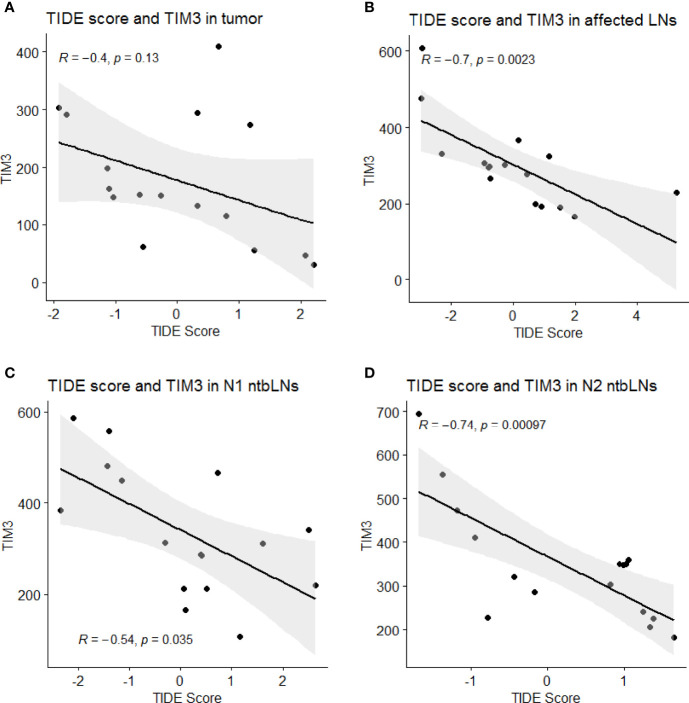
Scatterplots of TIDE scores and TIM-3 expression values in **(A)** tumor, **(B)** affected LNs, **(C)** N1 ntbLNs and **(D)** N2 ntbLNs with regression line and 95% confidence intervals.

We found TIM-3 to be inversely correlated with TIDE scores in affected LNs (r=-0.70, p=0.002), N1 ntbLNs (r=-0.61, p=0.01) and N2 ntbLNs (r=-0.74, p=0.001) (see **Figures 3A–D**). In addition, we found TIGIT expression in the tumor (r=-0.52, p=0.04) and FOXP3 in N1 ntbLNs (r=-0.56, p=0.03) to be correlated with TIDE scores (**Supplementary Table 3**).

**Figure 4:**
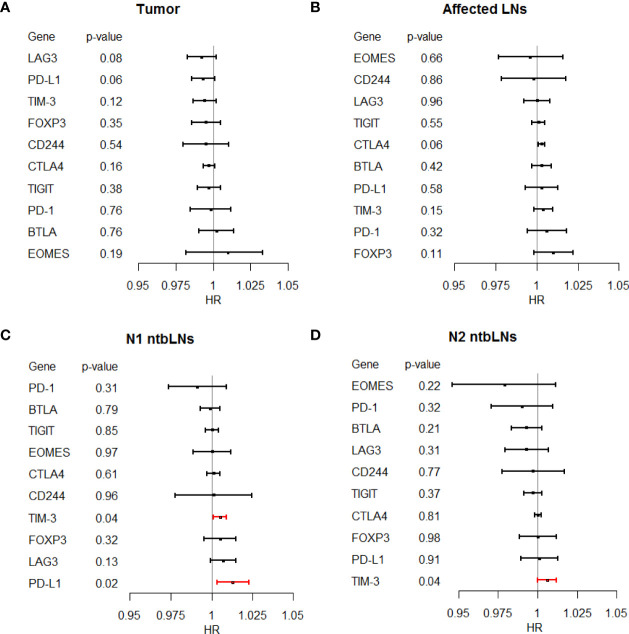
Forest plot of Hazard ratios from univariate Cox regression of T-cell exhaustion markers and overall survival in **(A)** tumor tissue, **(B)** affected LNs, **(C)** N1 ntbLNs and **(D)** N2 ntbLNs.

Expression levels of TIM-3 and PD-L1 in N1 and TIM-3 in N2 ntbLNs were associated with OS in univariate Cox regression (**Figures 4A–D**; **Supplementary Table 1**). We then also performed multivariate regression (**Table 3**). PD-L1 as well as TIM-3 were the only exhaustion markers to be selected for the final model for OS in more than one tissue (even though PD-L1 only reached a p-value of 0.06). The above data support our hypothesis that markers of immune cell exhaustion in ntbLNs may aid in the search for suitable biomarkers for immune exhaustion in NSCLC.

**Table 3:** Immune cell exhaustion markers selected for the final model of overall and progression-free survival using multivariate forward Cox regression.

Tissue	Type of survival	Variable	coef	SE	HR	p-value
Tumor	OS	PD-L1	0.003	0.002	1.003	0.062
AffLN	OS	CTLA4	0.003	0.001	1.003	0.059
N1 ntbLN	OS	TIM-3	0.005	0.003	1.005	0.064
		PD-L1	0.015	0.007	1.015	0.031
N2 ntbLN	OS	TIM-3	0.006	0.003	1.006	0.035
Tumor	PFS	Failed to converge				
AffLN		Failed to converge				
N1 ntbLN		Failed to converge				
N2 ntbLN		Failed to converge				

HR, Hazard Ratio; LN, Lymph node; ntb, non-tumor bearing; OS, Overall survival; PFS, Progression-free survival; SE, Standard error; TIDE, Tumor Immune Evasion and Dysfunction.

We also performed univariate Cox regression to determine factors which influence PFS. The results of the univariate regression analyses are displayed in **Supplementary Table 1**. Unfortunately, the multivariate regression models failed to converge, so no results could be obtained.

## Discussion

To our knowledge, this study presents the first analysis of immunosuppression and immune cell exhaustion in matched tumor, tumor-bearing LNs and ntb N1 and ntb N2 LNs in surgically treated NSCLC. We evaluated the predictive power of the TIDE score on survival in different tissues and showed differences in the relative abundance of CD8 cells in TIDE High and TIDE Low patients. In addition, we showed that exhaustion markers PD-L1 and TIM-3 are associated with survival even in early-stage NSCLC not treated with immunotherapy.

The TIDE algorithm was developed to assess the state of the immune system in tumors and to predict which tumors may respond to immunotherapy. It takes into account T-cell dysfunction as well as their exclusion from the tumor. Our results indicate that patients whose N1 ntbLNs and N2 ntbLNs show high TIDE levels have better overall survival than patients with low TIDE levels. Higher TIDE scores indicate a lack of immune evasion caused by high levels of tumor-infiltrating lymphocytes, activating immune cell signatures and permissive surrounding cellular milieu (mediated by cells such as macrophages and fibroblasts). This lack of immune suppression in regional lymph nodes points towards the fact that the immune system was able to retain at least some local control of the tumor. In this study, TIDE scores in N1 and N2 ntbLNs were a better indicator of survival than TIDE scores in the tumor. Since the patients in this study had early-stage NSCLC, it may be the case that their tumors had not yet developed the immunosuppressive environment that is typical of advanced tumors (13, 14). High TIDE scores (and therefore the presence of high numbers of functional cytotoxic lymphocytes) in N1 and N2 ntbLNs may indicate the recognition of the tumor by cytotoxic T-cells as their cognate antigen.

We found markers of immune exhaustion to be negatively correlated with TIDE scores. Since negative TIDE scores indicate the presence of dysfunctional lymphocytes, this dysfunction may be cause by an increased expression of exhaustion markers. However, markers of immune exhaustion such as TIM-3 can be expressed on multiple cell types such as dendritic cells, natural killer (NK) cells or CD8+ T-cells. This diverse pattern can complicate interpretation of results of immune exhaustion marker expression. It was shown that TIM-3 is an important gatekeepers of inflammasome regulation and TIM-3 deletion in dendritic cells led to an increase of anti-tumor activity. However, deletion of TIM-3 on CD4 or CD8 T-cells did not produce the same effect (15). To contrast this, an inducible liver cancer model showed that and TIM-3 expression on cytotoxic CD8 T-cells appeared early and led to loss of antitumor effector function (16). Taken together, the effects of immune exhaustion marker expression is dependent on many aspects such as tissue and cell type as well as timing during tumorigenesis. In the gene expression analysis presented here, we were not able to determine the cells of origin of exhaustion marker expression. However, we were able to show that an increased level of immune exhaustion marker expression overall led to shorter survival and are planning to further investigate the cells of origin.

TIM-3 is a marker of activated and subsequently of terminally exhausted CD8 cells, dendritic cells, NK cells and others and prevents the formation of long-lived memory cells. While the exact mechanism of TIM-3 mediated signalling is currently unknown, binding to one of its multiple interaction partners leads to increased suppressor function and reduces macrophage activation (17). Furthermore, TIM-3 inhibition leads to worsening of autoimmune inflammatory diseases such as inflammatory bowel disease and diabetes (18, 19). In this study, we found increased expression levels of TIM-3 in mediastinal and hilar lymph nodes to be associated with shorter overall survival. This parallels the findings of many other studies which have investigated TIM-3 levels in tumors (20–22). It was demonstrated that the tumor-draining lymph nodes of hepatocellular carcinoma acquire an immunosuppressive milieu (23). Taken together, these findings highlight the importance of assessing the state of the immune system in tumor-free local lymph nodes.

This study highlights the role of the immune cell dysfunction in surgically treated early-stage NSCLC patients. By analyzing tumor as well as tissue from tumor-bearing lymph nodes as well as tumor-free N1 and N2 lymph nodes, we were able to identify unique immunosuppressive signatures associated with survival. A limitation of this study is tumor microenvironment and lymph node heterogeneity. There is no consensus about which location in the tumor is best assessed for immune transcriptomics. In addition, there is a potential for sampling bias because of the histological sections that were used in the analysis. Furthermore, since we did not perform single-cell transcriptomics, it was not possible to assign altered expression levels to a cell type of origin.

## Conclusion

To our knowledge, this is the first study to perform immune transcriptomics in tumor and tumor bearing and non-tumor bearing regional LNs in NSCLC. We showed that immune exhaustion markers are associated with survival in this early-stage surgically treated cohort. Future clinical trials of exhaustion inhibitors should include ntbLNs to help identify patients most likely to benefit from adjuvant approaches and more in-depth postsurgical follow-up.

## Data Availability Statement

The datasets presented in this study can be found in online repositories. The names of the repositories and accession numbers/links can be found below: GEO, NCBI: GSE197929; Figshare: https://doi.org/10.6084/m9.figshare.18707639.

## Ethics Statement

The studies involving human participants were reviewed and approved by Ethics Committee of the Medical Faculty (LMU). The patients/participants provided their written informed consent to participate in this study.

## Author Contributions

LS: Conceptualization, Methodology, Data curation, Formal analysis, Roles/Writing - original draft, Writing - review & editing JKo: Conceptualization, Methodology, Roles/Writing - original draft, Writing - review & editing JW: Formal analysis, Roles/Writing - original draft, Writing - review & editing JKu: Data curation, Writing - review & editing JN: Methodology, Writing - review & editing DK-G: Conceptualization, Writing - review & editing RK: Data curation CS: Conceptualization, Methodology, Writing - review & editing AJ: Conceptualization, Writing - review & editing JB: Conceptualization, Writing - review & editing AT: Conceptualization, Methodology, Funding acquisition, Roles/Writing - original draft, Writing - review & editing. All authors contributed to the article and approved the submitted version.

## Conflict of Interest

The authors declare that the research was conducted in the absence of any commercial or financial relationships that could be construed as a potential conflict of interest.

## Publisher’s Note

All claims expressed in this article are solely those of the authors and do not necessarily represent those of their affiliated organizations, or those of the publisher, the editors and the reviewers. Any product that may be evaluated in this article, or claim that may be made by its manufacturer, is not guaranteed or endorsed by the publisher.
